# *ARF6s* Identification and Function Analysis Provide Insights Into Flower Development of *Punica granatum* L.

**DOI:** 10.3389/fpls.2022.833747

**Published:** 2022-03-07

**Authors:** Yujie Zhao, Yuying Wang, Xueqing Zhao, Ming Yan, Yuan Ren, Zhaohe Yuan

**Affiliations:** ^1^Co-innovation Center for Sustainable Forestry in Southern China, Nanjing Forestry University, Nanjing, China; ^2^College of Forestry, Nanjing Forestry University, Nanjing, China

**Keywords:** pomegranate, auxin response factor 6, expression analysis, PgmiR167, endogenous hormones, flower development

## Abstract

Based on the genome and small-RNA sequencing of pomegranate, miRNA167 and three target genes *PgARF6* were identified in “Taishanhong” genome. Three *PgARF6* genes and their corresponding protein sequences, expression patterns in pomegranate flower development and under exogenous hormones treatments were systematically analyzed in this paper. We found that PgARF6s are nuclear proteins with conserved structures. However, PgARF6s had different protein structures and expression profiles in pomegranate flower development. At the critical stages of pomegranate ovule sterility (8.1–14.0 mm), the expression levels of *PgARF6s* in bisexual flowers were lower than those in functional male flowers. Interestingly, *PgARF6c* expression level was significantly higher than *PgARF6a* and *PgARF6b*. Under the treatment of exogenous IBA and 6-BA, *PgARF6s* were down-regulated, and the expression of *PgARF6c* was significantly inhibited. PgmiR167a and PgmiR167d had the binding site on *PgARF6* genes sequences, and PgARF6a has the directly targeted regulatory relationship with PgmiR167a in pomegranate. At the critical stage of ovule development (8.1–12.0 mm), exogenous IBA and 6-BA promoted the content of GA and ZR accumulation, inhibited BR accumulation. There was a strong correlation between the expression of *PgARF6a* and *PgARF6b*. Under exogenous hormone treatment, the content of ZR, BR, GA, and ABA were negatively correlated with the expressions of *PgARF6* genes. However, JA was positively correlated with *PgARF6a* and *PgARF6c* under IBA treatment. Thus, our results provide new evidence for *PgARF6* genes involving in ovule sterility in pomegranate flowers.

## Introduction

Pomegranate, belonging to Lythraceae (Yuan et al., 2018), is rich in polyphenols such as punicalagin, ellagic acid, and other ellagitannin-based compounds (Johanningsmeier and Harris, 2011). Pomegranate is an excellent fruit tree with economic, nutritional, medicinal, ornamental, and ecological values (Yuan, 2015), therefore it is reputed as a “super fruit” (Teixeira da Silva et al., 2013). Pomegranate trees produce bisexual flower and functional male flower. Bisexual flower has an apparent tubular shape, and can bear a fruit as time advancement, whereas functional male flower is bell-shaped which usually drops and fails to set fruit. Studies have revealed that female sterility in functional male flowers is due to the abnormal ovule development failing to generate egg cells (Cai et al., 1993; Chen L. N. et al., 2017). When the vertical diameter of bisexual flowers is 5.1–10.0 mm, inner integument primordia are being form. When it is 10.1–13.0 mm, the outer integument primordia are formed in bisexual flowers, while the inner integument primordia are characterized in functional male flowers. When bisexual flowers gain their vertical diameter to 13.1–15.0 mm, the integument continues to enlarge, whereas the inner integument of functional male flowers is stagnated and the ovules display wilting (Chen L. N. et al., 2017). It can be concluded that it is an important stage for pomegranate ovule development when the vertical diameter of a flower bud is 5.1–15.0 mm.

Auxin is closely related to the differentiation of plant cells and vascular tissues, the morphogenesis of rhizomes, apical dominance, tropism, and response to external pressure (Guilfoyle and Hagen, 2007; Kumar et al., 2011; Yang et al., 2013). Auxin is a significant growth regulator which participates in the growth and development process of plants by changing the expression of early reactive genes, such as *Aux*/*IAA*, *GH3*, and *SAUR* gene families (Abel and Theologis, 1996; Wan et al., 2014; Roosjen et al., 2017). The promoter region of these genes contain one *cis*-acting element (TGTCTC) consisting of six bases, which is known as auxin response elements (AuxRE) (Guilfoyle et al., 1998). ARF (Auxin response factor) is a transcription factor that can specifically recognize and bind auxin response elements. ARF participates in auxin signal transduction by activating or inhibiting auxin response gene expression (Tiwari et al., 2003; Zhao et al., 2016; Zhang F. et al., 2020). Previous studies have shown that ARFs play independent roles in auxin signal transduction, and ARFs interact with *WUS*, *AG*, and *AP2* which are involved in regulating the morphogenesis of pistillate primordia (Liu X. G. et al., 2014). In *Arabidopsis thaliana* ARF family members, *AtARF1* and *AtARF2* are involved in regulation of pistil growth, flower organ development and wither (Ulmasov et al., 1997; Ellis et al., 2005). *AtARF3* and *AtARF4* participate in plant reproduction and vegetative growth (Sessions et al., 1997; Pekker et al., 2005). *AtARF5* plays a critical role in the formation of vascular tissue and the establishment of embryo development model (Hardtke and Berleth, 1998; Johnson and Douglas, 2007; Miyashima et al., 2013). *AtARF6* and *AtARF8* are suggested to be involved in regulating floral organ development and affect ovule development (Tian et al., 2004; Nagpal et al., 2005). *ARF6* and *ARF8* promote jasmonic acid (JA) production and flower maturation (Nagpal et al., 2005). *AtARF8* is involved in regulating hypocotyl elongation and root growth (Tian et al., 2004; Nagpal et al., 2005; Wu et al., 2006). *ATARF7*/*ATARF19*, *ATIAA1*, and *ATLBD16*/*18*/*29* are involved in the regulation network of lateral root development in *Arabidopsis thaliana* (Fukaki et al., 2005). In the *arf7*/*arf19*, the overexpression of *LBD16/ASL18* and *LBD29/ASL16* lead to the formation of lateral roots (Fukaki et al., 2005; Li et al., 2006; Okushima et al., 2007; Feng et al., 2012). In “Dabenzi” pomegranate genome, three *ARF6* homologous genes are identified, which might result from *ARF6* functional differentiation (Huang et al., 2019).

MiRNAs (microRNAs) are the class of important non-coding small RNAs, the length of which is 21–24 nt and play essential roles in regulating plant growth and development. The previous results demonstrate that miRNAs regulate the expressions of *ARF* family members. The expressions of *ARF10*, *ARF16*, and *ARF17* are regulated by miR160 (Damodharan et al., 2016), and the target genes of miR167 are *ARF6* and *ARF8* (Wu et al., 2006). miR167, together with *ARF6* and *ARF8*, is involved in regulating the development and maturity of pistil and stamen (Wu et al., 2006). Previous studies have shown that miR167 overexpression leads to reduced ovule maturity (Ru et al., 2006). By degrading *ARF8* mRNA and inhibiting translation of *ARF6* mRNA, miR167 regulate their expression and participate in the plant’s growth and development. In fact, miR167 define the expression regions of *ARF6* and *ARF8* in stamens and ovules, which ensure the normal development of sepals, petals, anthers, pistils, and ovules (Ru et al., 2006; Wu et al., 2006). Up to now, the roles of *ARF6* have been investigated in *Arabidopsis thaliana*, tomato (Tang, 2016), apple (Li H. F. et al., 2018), and strawberry (Wang, 2019). However, there is no relevant report on *ARF6* function in pomegranate flowers development. By integrating the genomic, transcriptomic and small-RNA sequencing data of “Taishanhong” pomegranate, *PgARF6s* were identified, and the targeted relationships between *ARF6* homologous genes and miR167 were preliminarily demonstrated in pomegranate. At the same time, the correlation between the expression of *PgARF6s* and endogenous hormone content was analyzed during pomegranate flower development. Our results laid the foundation for clarifying the function of *ARF6* in pomegranate flower development.

## Materials and Methods

### Plant Material and Treatments

Functional male flowers and bisexual flowers of pomegranate were categorized into eight stages according to their pistil vertical diameter (Zhao et al., 2020), that is 3.0–5.0 mm (P1), 5.1–8.0 mm (P2), 8.1–10.0 mm (P3), 10.1–12.0 mm (P4), 12.1–14.0 mm (P5), 14.1–16.0 mm (P6), 16.1–18.0 mm (P7), and 18.1–20.0 mm (P8). The flower buds were collected at the Baima Base for Teaching and Scientific Research of Nanjing Forestry University. Three biological repeats of samples from each stage were prepared, frozen with liquid nitrogen and stored in the −78°C refrigerator for subsequent use.

Nine six-year-old “‘Taishanhong”’ plants were treated with 20.0 mg/L IBA and 100.0 mg/L 6-BA, respectively (Chen et al., 2020). After 1one week, functional male flowers and bisexual flowers were collected as test samples. The control samples were treated with clear water. The determination method of endogenous hormone content was described elsewhere (Zhao et al., 2021).

### Identification and Bioinformatics Analysis of PgARF6s in Pomegranate

Firstly, using the published “Taishanhong” pomegranate genome data (Yuan et al., 2018) and ARF (PF06507) data in the Pfam database, the hmmbuild model was adopted in the HMMER 3.0 software package, and the hmmsearch program was further used to screen candidate ARF protein sequences (*e*-value < 1e^–5^, identity > 50%). ARF protein sequences in plant transcription factor database were used as queries to perform BLAST against the pomegranate genome database (*e*-value < 1e^–5^, identity > 50%), duplication was removed and ARF protein sequences were screened and selected. Then, the CDD^1^ and SMART^2^ were used to detect protein structure domains of the candidate sequences. The exon-intron structures of *PgARF6* genes were displayed using Gene Structure Display Server 2.0.^3^ Motifs of *PgARF6s* nucleic acids sequences were identified using MEME^4^ with default parameter. Phyre 2.0 was used to predict ARF6 proteins structures.

*Eucalyptus grandis* (Eucgr), *Populus euphratica* (Pop), and *Carica papaya* (Capa) protein sequences were downloaded from plant transcription factor database, and *Arabidopsis thaliana* (AT) protein sequences were downloaded from TAIR database. The ARF phylogenetic tree of candidate proteins of *Eucalyptus grandis*, *Populus euphratica*, *Carica papaya*, and *Arabidopsis thaliana* were constructed on the online website.^5^ The amino acid sequences of PgARF6s were aligned by ClustalX. The evolutionary tree of species was constructed with MEGA 7.0 software, and plants protein sequences were downloaded from NCBI database.

PgARF6 proteins subcellular localization was predicted by Cell-PLoc 2.0.^6^ At the same time, the String (version 11.5) was used to predict the PgARF6 plant proteins interaction network, and the number of organism was set as “*Arabidopsis thaliana*.”

To examine expression patterns of *PgARF6s* in different pomegranate tissues, the published transcriptome data (Chen L. N. et al., 2017; Qin et al., 2017) were download for expression analysis (Supplementary Table 1). Firstly, all RNA-Seq data were qualitatively controlled by fastp to obtain cleaned reads. Then, the pomegranate transcriptome data were analyzed according to the method reported by Zhao et al. (2020). Finally, the heatmap of *PgARF6s* transcription levels was drawn.

### RNA Extraction, cDNA Synthesis and Gene Cloning

Total RNAs were extracted from the flower buds of pomegranate using the BioTeke plant total RNA extraction kit. The integrity and concentration of RNA were detected by agar-gel electrophoresis and UV-visible spectrophotometer ND-2000 (Thermo Fisher Scientific Inc., United States). cDNA was prepared by reverse transcription kit (PrimeScript™RT reagent Kit with gDNA Eraser, TaKaRa).

The CDS-full-length of *PgARF6a*, *PgARF6b*, and *PgARF6c* were cloned by homologous cloning. The CDS of *PgARF6* genes were extracted from the genome data of “Taishanhong” pomegranate, and Oligo 7.0 was used to design the gene clone primers (Table 1). A high-fidelity PCR reaction was carried out using the cDNA of mixed flowers samples as a template. The reaction conditions were as follows: first 98°C for 2 min; then 98°C for 15 s, 58°C for 15 s, 72°C for 1 min, and 30 cycles; last 72°C for 5 min. Then, the recovered PCR products were sent to Shanghai Sangon Biological Company for sequencing.

**TABLE 1:** Primers for the gene cloning, subcellular localization, and expression analysis.

Primer	Primer sequence	Annotation	Length of product
*PgARF6a*	F: ATGAGGCTTTCTTCTGCTT; R: TCAGTAATCGAGGGACCCC	Gene clone	2,700
*PgARF6b*	F: ATGAGACTGTCTCCAGCTG; R: TTAGTAATCAAGAGATCCT	Gene clone	2,901
*PgARF6c*	F: ATGAGGCTCTCTTCGCCTG; R: TCAGTAGTCAAGAGACCCT	Gene clone	2,607
*PgARF6a*	F: GAGAACACGGGGGACTCTAGAATGAGGCTTTCTTCTGCTT; R: GCCCTTGCTCACCATGGATCCGTAATCGAGGGACCCCACG	Subcellular localization	2,700
*PgARF6b*	F: GAGAACACGGGGGACTCTAGAATGAGACTGTCTCCAGCTG; R: GCCCTTGCTCACCATGGATCCGTAATCAAGAGATCCTCTT	Subcellular localization	2,901
*PgARF6c*	F: GAGAACACGGGGGACTCTAGAATGAGGCTCTCTTCGCCTG; R: GCCCTTGCTCACCATGGATCCGTAGTCAAGAGACCCTATG	Subcellular localization	2,607
*qRTPgARF6a*	F: CCTCCTTTGTTCTTTGCC; R: CATCTTGGGTGAGACGTTAT	Gene expression	200
*qRTPgARF6b*	F: CTGTCGGAACCCGTGTAGT; R: CAGGGTCATCTGAGCATAAA	Gene expression	200
*qRTPgARF6c*	F: CAACCCAAGGGCAAGTCC; R: AGCATCCTGAACCGCATT	Gene expression	200
*PgActin*	F: AGTCCTCTTCCAGCCATCTC; R: CACTGAGCACAATGTTTCCA	Gene expression	200

### PgARF6s Subcellular Localization

The linearized pBI121 vector was obtained by double digestion of restriction enzymes *Xba*I and *Bam*HI. Homologous arm primers were designed by adding restriction site sequences at both ends of the target genes (Table 1). According to ClonExpress^®^II One Step Cloning Kit (Vazyme Biotech Co., Ltd.), *PgARF6s* were connected with the modified pBI121-GFP vector, and the recombinant vectors were transferred into *DH5*α. Then, the constructed vectors plasmids were extracted and transferred into Agrobacterium GV1301 competent cells by the freeze-thaw method.

Monoclonal colonies were selected and inoculated into a 20 mL liquid medium. The bacteria solution was 5,000 r/min, centrifuged for 5 min to collect the bacteria, and then added with 20 mL leaching solution to re-suspend the bacteria. The suspension bacteria were injected into tobacco. After injection, tobacco returned to normal growth after dark culture for 24 h, and fluorescence observation was performed 1–2 days later.

### Expression Analysis of PgARF6s

The expression patterns of *PgARF6s* were studied using bisexual flowers and functional male flowers from different development stages. According to the CDS of *PgARF6s*, quantitative realtime PCR (qRT-PCR) primers were designed (Table 1). qRT-PCR methods had been described previously (Zhao et al., 2020), and the data were analyzed by 2^–ΔΔ^*CT* method.

### Prediction of PgmiR167 Target Sites of PgARF6s in Pomegranate

The nucleotide sequences of *PgARF6s* were analyzed by psRNATarget online software to predict the target site of PgmiR167.

Functional male flowers and bisexual flowers with the vertical diameter of 5.0–10.0, 10.1–13.0, and 13.1–18.0 mm were selected and sent to Novogene Company (Beijing) for transcriptome (PRJNA754480) and small-RNA sequencing (PRJNA793612). Sequencing libraries were generated using NEBNext^®^ Ultra™ RNA Library Prep Kit for Illumina^®^ (NEB, United States). Small RNA libraries were generated using Small RNA Sample Pre Kit. Index of pomegranate reference genome (ASM220158v1) was built using Hisat2 v2.0.5 and clean reads were aligned to pomegranate genome using Hisat2 v2.0.5. featureCounts v1.5.0-p3 was used to count the reads numbers mapped to each gene. Then, FPKM of each gene was calculated based on the length of the gene and reads count mapped to this gene. FPKMs of *PgARF6s* and PgmiR167 were converted into Log_2_(FPKM + 1).

### Statistical Analysis

All experimental data were analyzed using SPSS software (22.0, United States). The data were expressed as the means and standard deviations (mean ± SD). Different letters were the significant difference among developmental stages for flower, based on Duncan’s Test at the *p* < 0.05 level.

## Results

### Identification and Bioinformatics Analysis of Pomegranate ARF6

**FIGURE 1:**
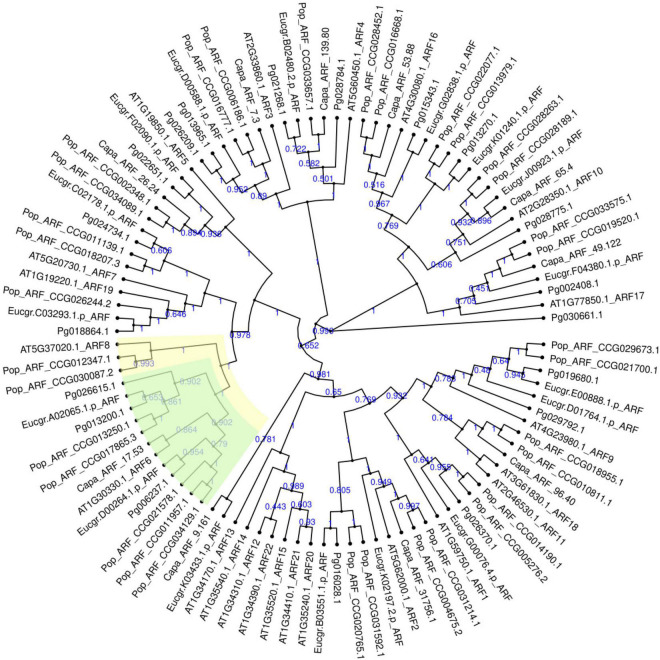
Phylogenetic tree of ARF gene family in *Punica granatum* (Pg), *Eucalyptus grandis* (Eucgr), *Populus euphratica* (Pop), *Carica papaya* (Capa), and *Arabidopsis thaliana* (AT).

A total of 19 ARF genes were identified from the genome of “Taishanhong” pomegranate using two methods of local blast and hmmsearch model. All candidate proteins were confirmed to belong to ARF family (Supplementary Table 2). CDD and SMART conserved domains showed that Pg006237.1, Pg013200.1, and Pg026615.1 have typical B3, Auxin_resp, and Aux_IAA conserved domains. Pg006237.1, Pg013200.1, and Pg026615.1 were renamed as PgARF6a, PgARF6b, and PgARF6c for convenience in subsequent analysis, based on their potential orthologs proteins in *Arabidopsis thaliana* (Figure 1).

**FIGURE 2:**
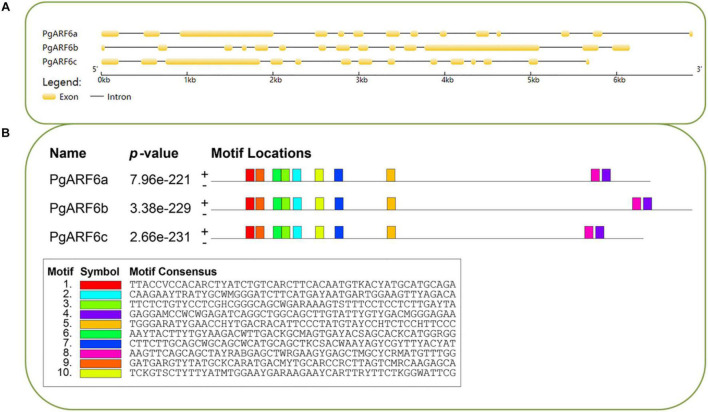
Basic information of *PgARF6* sequences in pomegranate. **(A)** Gene structure of *PgARF6s*, **(B)** conservative nucleic acid motifs of *PgARF6s*.

Gene structures were investigated on gene structure display server (GSDS). *PgARF6a*, *PgARF6b*, and *PgARF6c* contained 14 exons, indicated that the gene structure with 13 introns was the main form of *PgARF6s*. The results of conservative motifs showed similar conservative motif in *PgARF6* genes. These results indicated that the motif and gene structure of *PgARF6s* were highly consistent and conservative (Figure 2).

**FIGURE 3:**
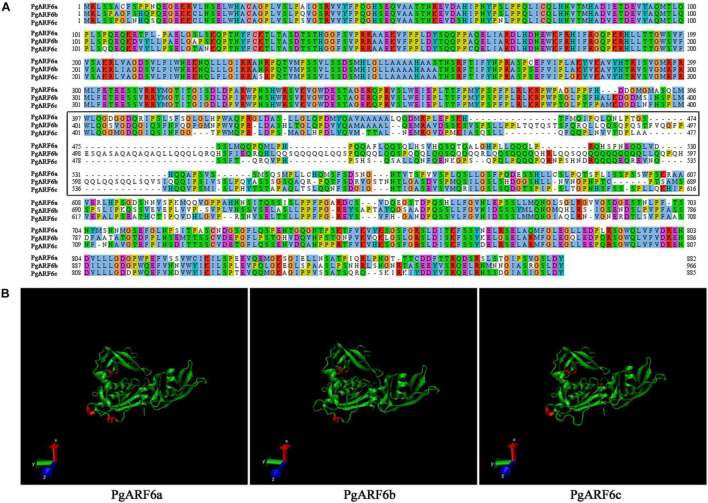
**(A)** Sequences alignment of PgARF6s. **(B)** The tertiary structure of PgARF6s.

To better understand protein structure and functional information of pomegranate ARF6s, we performed a systematic bioinformatics analysis of PgARF6 protein sequences. The multi-sequence alignment analysis showed that three PgARF6 homologous proteins had long conserved sequences in amino acid composition, but protein sequences were different at the sites 387–607 (PgARF6a) (Figure 3A). Proteins insertions were observed at sites 387–389 (AMK) of PgARF6c and sites 492–519 (FVQGFPESQASAQAQAQAQLLQQQLQRQ) of PgARF6b, and proteins deletions were found at sites 419–421 (LGL) of PgARF6c and sites 678–683 (SSPSSW) of PgARF6b. Then, tertiary structure analysis of PgARF6 proteins showed that the structure of ARF6 proteins were highly similar, but the 3_10_Helix (red) structures were located differently in PgARF6a, PgARF6b, and PgARF6c (Figure 3B). We found that the tertiary structure of PgARF6b and PgARF6c were derived from PgARF6a, and the structure was also different between PgARF6b and PgARF6c.

**FIGURE 4:**
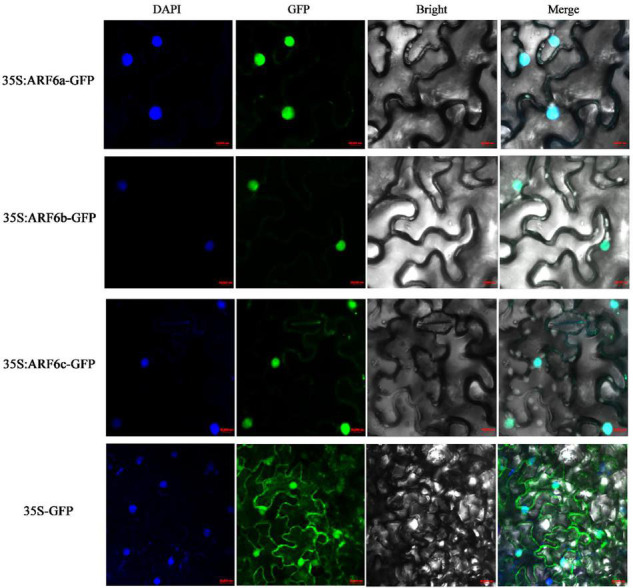
Subcellular localization of PgARF6s-GFP fusion in tobacco.

The Cell-PLoc 2.0 prediction results showed that PgARF6a, PgARF6b, and PgARF6c were located in the cell nucleus. CDSs of *PgARF6a*, *PgARF6b*, and *PgARF6c* were cloned (Supplementary Figure 1) and submitted to the NCBI database (GenBank number: OM179998, OM179999, OM180000). Then, GFP fluorescence signals of the control were observed in both cell membrane and nucleus, while that of PgARF6-GFP were only observed in the nucleus (Figure 4), indicating that PgARF6 proteins were localized in cell nucleus and had the subcellular localization characteristics of typical transcription factors.

Protein-protein interaction analysis results (Supplementary Figure 2) showed that PgARF6a, PgARF6b, and PgARF6c had the highest similarity with AtARF6 (*E*-value 0.0e^+00^, 2.2e^–305^, 6.1e^–302^, respectively), suggesting that PgARF6s might have the same function with AtARF6. ARF6 with Aux/IAA proteins fuse heterodimers that is involved in regulating auxin response gene expression, stamen and gynoecium maturation (Piya et al., 2014). ARF6 promotes JA production, partially redundant with ARF8. ARF6, BZR1, and BEE 2 positively regulate brassinosteroid (BR) signaling (Li Q. F. et al., 2018; Tian et al., 2018). IAA8, IAA19, and IAA28 are short-lived transcriptional factors that inhibit the expression of auxin response genes at low auxin concentrations (Piya et al., 2014). The repressors are due to their binding to AuxRE in the ARF promoter region (Hussain et al., 2020).

To understand the relationships between ARF6 proteins in pomegranate and other plants, we conducted phylogenetic analyses of known *ARF6* using amino acid sequences. It was found that PgARF6 proteins were highly similar with other plants, indicating that the amino acid sequences of ARF6 in different plants were relatively conservative (Supplementary Figure 3). Phylogenetic analyses showed that PgARF6s could successfully cluster with *Arabidopsis thaliana*, *Solanum lycopersicum*, *Citrus clementina*, and *Carica papaya* (Supplementary Figure 4). PgARF6 proteins had a far evolutionary relationship with ARF6 of *Zea mays*, *Ananas comosus*, and *Malus domestica*, whereas PgARF6 proteins clustered more closely to *Solanum lycopersicum*, *Morus notabilis*, and *Carica papaya*.

**FIGURE 5:**
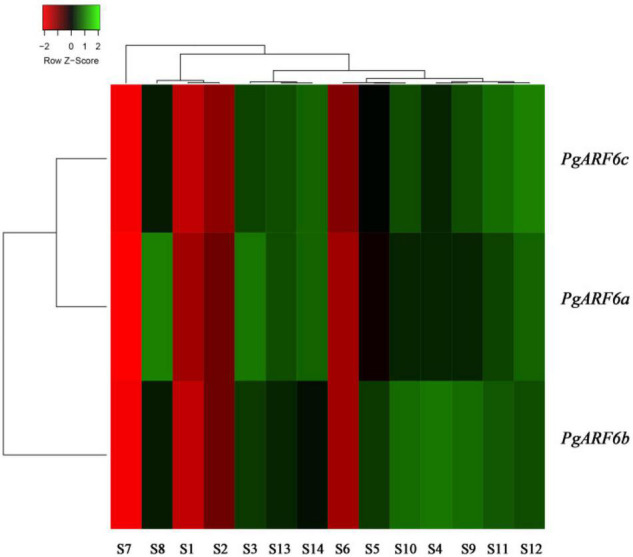
*PgARF6* expression heatmap in different organs of pomegranate. S1, “Baiyushizi” inner seed coat; S2, “Tunisiruanzi” inner seed coat; S3, flower; S4, leaf; S5, root; S6, inner seed coat; S7, outer seed coat; S8, pericarp; S9, bisexual flower I; S10, functional male flower I; S11, bisexual flower II; S12, functional male flower II; S13, bisexual flower III; S14, functional male flower III (S3–S8 were “Dabenzi,” S9–S14 were the pistil of “Tunisiruanzi”).

In order to uncover the roles of *PgARF6* genes in pomegranate different organs, *PgARF6s* expression levels in different tissues were analyzed using transcriptome data. The results showed that *PgARF6s* were expressed in leaf, root, flower, fruit, endocarp, and pericarp (Figure 5). However, the results revealed the differences of expression levels for three *PgARF6* genes. We found that *PgARF6s* were weakly expressed in seed coat, whereas *PgARF6s* were highly expressed in flowers, indicating that *PgARF6s* might be involved in pomegranate flower organ development.

### Expression Profiles of PgARF6s During Flower Development in Pomegranate

**FIGURE 6:**
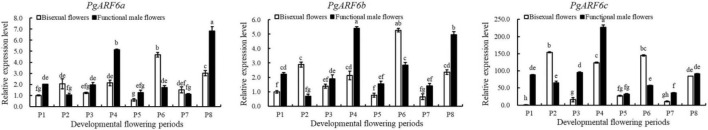
Expression of *PgARF6s* transcripts during pomegranate flowers development.

To explore the putative function of *PgARF6s* in floral organ development, the expression patterns of *PgARF6s* in bisexual flowers and functional male flowers were analyzed by qRT-PCR (Figure 6). The expression level of *PgARF6a* in bisexual flowers was the highest at P6, which was 9 times than that at P5 and 4.6 times than that at P1. At the critical stage of ovule primordia formed (5.1–8.0 mm, P2), *PgARF6a* transcriptional level in bisexual flowers was significantly higher than that in functional male flowers. During flowers development, the expression pattern of *PgARF6b* was consistent with that of *PgARF6a*. At the critical stage of ovule sterility (8.1–12.0 mm, P3–P4), transcriptional levels of *PgARF6a* and *PgARF6b* in bisexual flowers were significantly lower than that in functional male flowers.

The expression level of *PgARF6c* in bisexual flowers was the highest at the P2 stage, which were 9.5 times than that of P3 and 15 times than that of P7. During functional male flower development stages, *PgARF6c* transcriptional level was the highest at the P4 stage, which was 7.1 times higher than that at P5 stage. At the period of ovule primordia formed (5.1–8.0 mm, P2), the expression levels of *PgARF6a*, *PgARF6b*, and *PgARF6c* in bisexual flowers were higher compared with that in functional male flowers. These results showed that *PgARF6s* expression levels in bisexual flowers were significantly lower than that in functional male flowers at the critical stage of ovule sterility (8.1–12.0 mm, P3–P4), indicating that *PgARF6s* was involved in ovule sterility developing into functional male flower.

### The Relationships of PgmiR167 Targeted PgARF6s in Pomegranate

To investigate the targeted relationships between *PgARF6s* and PgmiR167, we predicted the target sites of PgmiR167 based on the *PgARF6* sequences (Table 2). There were two base differences between PgmiR167a and PgmiR167d that A of PgmiR167a was replaced by GG. PgmiR167a had the same sequence of binding sites on *PgARF6*, and there was only one binding site. PgmiR167d and PgmiR167a had a similar situation. The results indicated that *PgARF6s* genes were targets of PgmiR167a and PgmiR167d, suggesting that regulation of ARF6s by miR167 was conserved between pomegranate, tomato (Liu N. et al., 2014) and *Arabidopsis* (Wu et al., 2006).

**TABLE 2:** Prediction of targeting relationship between *PgARF6s* and PgmiR167 in pomegranate.

miRNA	Target gene	Target start	Target end	MiRNA aligned fragment	Target aligned fragment
PgmiR167a	*PgARF6a*	2,370	2,390	UGAAGCUGCCAGCAUGAUCUA	GAGAUCAGGCUGGCAGCUUGU
PgmiR167a	*PgARF6b*	2,619	2,639	UGAAGCUGCCAGCAUGAUCUA	GAGAUCAGGCUGGCAGCUUGU
PgmiR167a	*PgARF6c*	2,382	2,402	UGAAGCUGCCAGCAUGAUCUA	GAGAUCAGGCUGGCAGCUUGU
PgmiR167d	*PgARF6b*	2,618	2,639	UGAAGCUGCCAGCAUGAUCUGG	AGAGAUCAGGCUGGCAGCUUGU
PgmiR167d	*PgARF6c*	2,381	2,402	UGAAGCUGCCAGCAUGAUCUGG	AGAGAUCAGGCUGGCAGCUUGU
PgmiR167d	*PgARF6a*	2,369	2,390	UGAAGCUGCCAGCAUGAUCUGG	UGAGAUCAGGCUGGCAGCUUGU

**FIGURE 7:**
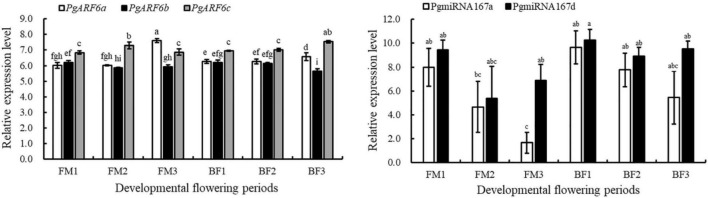
Transcriptome and small-RNA sequencing results of *PgARF6*-PgmiR167 expression analysis.

To determine the relationships between PgmiR167 and *PgARF6* genes at transcript level, we further analyzed the expression profiles of *PgARF6* and PgmiR167 based on data of transcriptome and small-RNA sequencing (Figure 7). During functional male flower development, the expression level of *PgARF6a* increased, but PgmiR167a expression level gradually decreased. During bisexual flowers development, the expression of *PgARF6a* gradually increased, whereas the expression of PgmiR167a decreased. The transcriptional level of PgmiR167a gradually decreased in functional male flowers and bisexual flowers, while the expression level of PgmiR167d decreased then increased. The expression levels of *PgARF6b* slowly decreased in bisexual flowers and functional male flowers, while an opposite trend of *PgARF6a* was observed. The transcriptional level of *PgARF6c* was firstly increased and then decreased in functional male flowers, while gradually increased in bisexual flowers. These results indicated that PgmiR167a directly regulated *PgARF6a* in pomegranate flowers.

### Expression of PgARF6 Genes in Response to 6-BA Treatment

**FIGURE 8:**
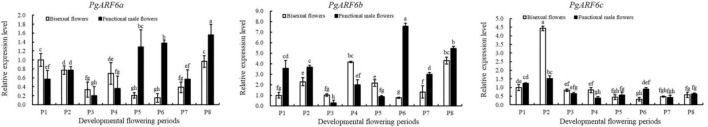
Expression analyses of *PgARF6s* under 6-BA treatment.

To determine the response of PgARF6 genes to exogenous cytokinin stimuli, their expression patterns in pomegranate flowers were investigated after 6-BA treatments (Figure 8). Under 6-BA treatment, the expression level of *PgARF6a* in bisexual flowers was higher than that in functional male flowers at the critical stage of ovule development (3.0–12.0 mm, P1–P4). The expression level of *PgARF6a* in bisexual flowers was 1.9 times and 1.7 times higher than that in functional male flowers at P4 and P3 stage, respectively. Whereas *PgARF6a* expression level in bisexual flowers was lower than that in functional male flowers at the development period of organ maturation (P5–P8). After being induced by exogenous 6-BA, *PgARF6b* transcriptional level in bisexual flowers was lower than that in functional male flowers at the period of ovule primordia development (3.0–8.0 mm, P1–P2). At the critical stage of ovule sterility (8.1–12.0 mm, P3–P4), *PgARF6b* transcriptional level in bisexual flowers was higher than that in functional male flowers. The transcriptional level of *PgARF6b* in bisexual flowers was 3.7, 2.1, and 2.5 times higher than that in functional male flowers at P3, P4, and P5, respectively.

Under 6-BA treatment, *PgARF6c* expression level in bisexual flowers was higher than that in functional male flowers at the critical stage of ovule development (5.1–12.0 mm, P2–P4). *PgARF6c* expression level in bisexual flowers was 2.9 times higher than that in functional male flowers at P2. The level of *PgARF6c* was down-regulated after P2 period.

### Response of Endogenous Hormone in Pomegranate Flowers to Exogenous 6-BA Treatment

**FIGURE 9:**
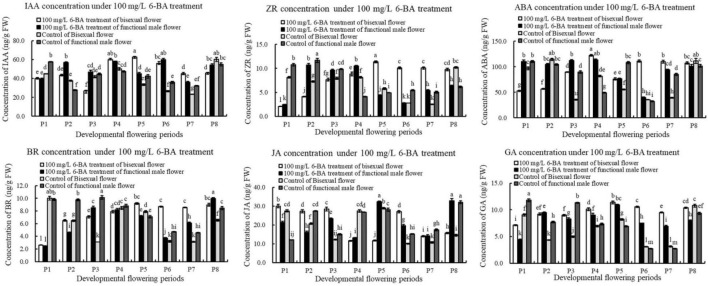
Endogenous hormone in pomegranate flowers under 6-BA treatment.

The content of IAA under exogenous 6-BA treatment decreased, increased and then decreased during bisexual flowers development (Figure 9). The content of IAA in the treatment was higher than that in the control at P2 and P4–P5 stages of the critical stage of ovules development. During functional male flowers development, the content of IAA induced by exogenous 6-BA was higher than that in the control, but lower than that in the control at P1.

Under 6-BA treatment, ZR content in bisexual flowers gradually increased, but there were no significant differences among P6, P7, and P8. At the critical stage of ovule development in bisexual flower (P1–P3), the content of IAA in the treatment was higher compared with that in the control. During the development of functional male flowers, the change pattern of ZR content in the treatment was consistent with the control. During P1–P3 stages of functional male flower, the content of IAA in the treatment was lower than that in the control. Under 6-BA treatment, the content of ZR in bisexual flowers was lower than that in functional male flowers at P1–P4 stages, which was consistent with the control. However, the content of ZR in bisexual flowers was higher than that in functional male flowers at P5–P8 stages (Figure 9).

Under 6-BA treatment, ABA content in functional male flowers decreased then increased. ABA content in bisexual flowers was lower than that in functional male flowers at P1–P3 stages. During the bisexual flowers development, ABA content in the treatment was lower than that in the control at P1–P2 stages, but higher than that in the control at P3–P5 stages. During the functional male flowers development, ABA content in the treatment was higher than that in the control at P3–P4 stages (Figure 9).

**FIGURE 10:**
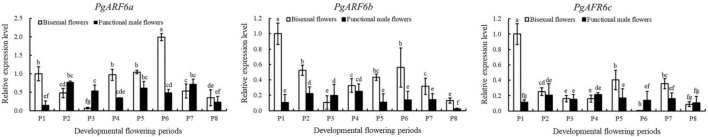
Expression analyses of *PgARF6s* under IBA treatment.

Under 6-BA treatment, BR content in bisexual flowers showed a gradually increasing trend, the content of BR in functional male flowers showed the increasing- decreasing- increasing trend (Figure 10). Under 6-BA treatment, BR content in bisexual flowers was higher than that in functional male flowers at P1–P2 and P5–P7 stages, but lower at P3–P4 stages. At P1–P6 stages of the functional male flowers development, the content of BR in the treatment was lower compared with that in the control.

After the induction of exogenous 6-BA, the content of JA exhibited a trend of firstly decreased, next increased, and then decreased during bisexual flowers development (Figure 9). Under 6-BA treatment, JA content in bisexual flowers was higher compared with functional male flowers at P1–P3 stages, whereas bisexual flowers were lower than functional male flowers at P4–P5 stages. At P1–P3 stage of the bisexual flowers development, the content of JA in the treatment was higher than that in the control.

Under 6-BA treatment, GA content in bisexual flowers increased then decreased, this change pattern was consistent with that in functional male flowers (Figure 9). Under 6-BA treatment, the concentration of GA in bisexual flowers was higher compared with functional male flowers (except P2). During P2–P7 stage for the bisexual flowers, a higher content of GA in the treatment was observed than that in the control. At P2 and P4–P7 stages for the functional male flowers, the content of GA in the treatment was higher than that in the control.

### Expression of PgARF6 Genes in Response to IBA Treatment

To determine the response of *PgARF6* genes to exogenous IBA, the expression patterns of *PgARF6* in pomegranate flowers after IBA treatment were investigated using qRT-PCR. Under IBA treatment, the transcriptional level of *PgARF6a* in bisexual flowers was lower compared with that in functional male flowers at ovule development P2–P3 stages, and bisexual flowers was 7.7 times lower than functional male flowers at P3 (Figure 10). *PgARF6a* expression level of bisexual flowers was higher than functional male flowers at P4–P6. The transcriptional level of bisexual flowers was 2.8, 1.7, and 4.0 times higher than functional male flowers at P4, P5, and P6, respectively.

Induced by exogenous 6-BA, the expression level of *PgARF6b* in bisexual flowers was higher compared with that in functional male flowers during the whole development process (except P3). At the P3 stage, *PgARF6b* transcriptional level in bisexual flowers was 1.9 times lower than that in functional male flowers. At ovule development P2, P4, and P5 stages, *PgARF6b* expression level in bisexual flowers was 2.4, 1.32, and 3.9 times higher than that in functional male flowers, respectively.

Under 6-BA treatment, the transcriptional level of *PgARF6c* in bisexual flowers was higher than that in functional male flowers at the critical stage of ovule development (3.0–10.0 mm, P1–P3). At P4 stage of ovule sterility, *PgARF6c* expression level in bisexual flowers was 1.3 times lower than that in functional male flowers. At P5 stage, *PgARF6c* expression level in bisexual flowers was 2.4 times higher than functional male flowers.

### Response of Endogenous Hormone in Pomegranate Flowers to Exogenous IBA Treatment

**FIGURE 11:**
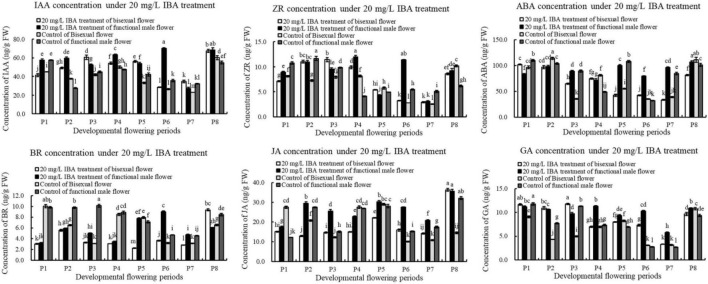
Endogenous hormone in pomegranate flowers under IBA treatment.

We further analyzed the content changes of endogenous hormone in pomegranate flower development under the treatment of exogenous IBA (Figure 11). Under IBA treatment, the content of IAA in bisexual flowers firstly increased, decreased and then increased during flower development. The concentration of IAA in bisexual flowers was lower than that in functional male flowers at P1–P2 and P4 stages, but higher than that in functional male flowers at P3, P5, and P7 stages. At the critical period of ovule development (P2–P5), the content of IAA in the treatment was higher compared with that in the control.

Under IBA treatment, ZR content in bisexual flowers firstly increased, decreased and then increased (Figure 11). Under IBA treatment, the concentration of ZR in bisexual flowers was lower than that in functional male flowers at P1–P2 and P4–P5 stages, but higher at P3 and P5 stages. During the bisexual flowers development, ZR content in the treatment was higher than that in the control at the critical stage of ovule development (P2–P4), but lower at P1 and P5 stages. During the functional male flowers development, ZR concentration in the treatment was higher than that in the control at P1–P3 stages, whereas at P4–P5 stages.

After external IBA induction, ABA content decreased then increased (P8) during bisexual flowers development (Figure 11). Under IBA treatment, the content of ABA in bisexual flowers was lower than that in functional male flowers at P2–P3 and P5–P8 stages, but higher at P1 and P4 stages. During the P2–P4 stages of functional male flowers ovule abortion, the concentration of endogenous ABA showed a decreasing trend.

During the development of the bisexual flowers, the content of BR under IBA treatment firstly increased, decreased and then increased (Figure 11). Under IBA treatment, the content of BR in bisexual flowers was lower compared with that in functional male flowers, except P8. At P1–P5 stages of bisexual flowers ovule development, the concentration of BR in the treatment was lower than that in the control. During the functional male flowers development, BR content in the treatment was lower than that in the control at P1–P4 stages, but higher than that in the control at P5–P7 stages.

Under IBA treatment, the content of JA in bisexual flowers was lower compared with functional male flowers, except P8 (Figure 11). During the bisexual flowers development, JA content in the treatment was lower than that in the control at P1–P2 and P4–P5 stages, whereas BR content in the treatment was higher than that in the control at P3 and P6–P8 stages. During the functional male flowers development, JA concentration in the treatment was higher than that in the control, but lower at P4 stage.

Under exogenous IBA treatment, the content of GA in bisexual flowers was higher than that in functional male flowers at P1–P4 and P6–P7 stages, but lower at P5 and P8 (Figure 11). At the critical stage of ovule development in bisexual flower (3.0–12.0 mm, P1–P4), the concentration of GA in the treatment was higher compared with that in the control. During the functional male flowers development, GA content in treatment was higher than that in the control, except P1 and P3 stages.

### The Correlation Between Endogenous Hormones and PgARF6s Expression Levels After Exogenous IBA and 6-BA Treatment

**FIGURE 12:**
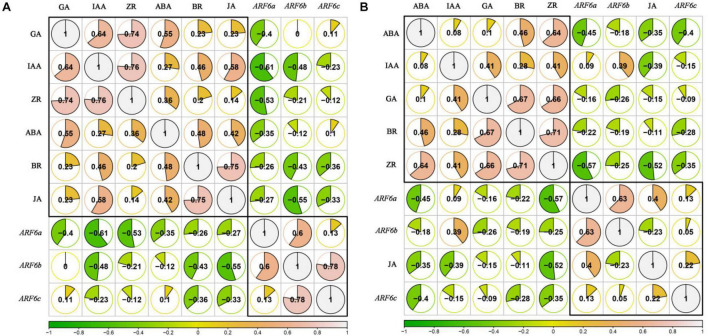
**(A)** The correlation between endogenous hormones and *PgARF6* genes expression after IBA treatment. **(B)** The correlation between endogenous hormones and *PgARF6* genes expression after 6-BA treatment.

In order to analyze the relationships between endogenous hormones content (IAA, ZR, BR, JA, GA, and ABA) and *PgARF6s* expression levels in pomegranate flowers development after exogenous IBA and 6-BA treatment, the correlation coefficient was calculated by R-rcorr and visualized by R-corrplot. As illustrated in Figure 12A. When treated with IBA, the endogenous BR, JA, IAA, and ZR levels were negatively correlated with the expressions of *PgARF6a*, *PgARF6b*, and *PgARF6c*. GA content was negatively correlated with *PgARF6a* expression, but positively correlated with the expression levels of *PgARF6b* and *PgARF6c*. ABA content was negatively correlated with the expression levels of *PgARF6a* and *PgARF6b*, but slightly positively correlated with *PgARF6c*. The pairwise positive correlation was observed between the endogenous IAA, ZR, BR, JA, GA, and ABA. There were the significantly positive correlation between GA, IAA, and ZR. JA was significantly positively correlated with BR. *PgARF6b* was significantly positively correlated with *PgARF6a* and *PgARF6c*, but the positive correlation between *PgARF6a* and *PgARF6c* was not significant.

Figure 12B showed the correlation between the content of endogenous hormones and the expression levels of *PgARF6s* after exogenous 6-AB treatment. The contents of endogenous BR, ZR, GA, and ABA were negatively correlated with the expression levels of *PgARF6s*. IAA content was negatively correlated with *PgARF6c* expression level, but positively correlated with *ARF6a* and *PgARF6b* transcriptional levels. JA content was positively correlated with the expression levels *PgARF6a* and *PgARF6c*, but negatively correlated with *PgARF6b*. There were the positive correlation between the endogenous hormones ABA, IAA, ZR, GA, and BR. There was a significantly positive correlation between GA, BR, and ZR. ABA was significantly positive with ZR, and JA had a negative correlation with other endogenous hormones. *PgARF6a* was significantly positively correlated with *PgARF6b*, but *PgARF6c* was positively correlated with *PgARF6a* and *PgARF6b*. These results showed that endogenous hormones content and *PgARF6* genes expressions interact with each other, indicating a complex process required multiple factors to regulate pomegranate flower development.

## Discussion

### PgARF6s Identification and Bioinformatics Analysis

ARF is involved in regulating multiple processes in plants and plays crucial roles in growth and development (Yao et al., 2019; Zheng et al., 2019; Mao et al., 2020). *ARFs* play an independent role in auxin signal transduction and participate in regulating embryogenesis, flowering and fruit-setting (Wu et al., 2011; Shi et al., 2014). Previous researches have shown that *ARFs* interact with *AG*, *AP2*, and miRNAs to participate in the morphogenesis of pistil (Liu N. et al., 2014; Liu X. G. et al., 2014). In our study, three *PgARF6* genes were identified and cloned from the “Tanshanhong” pomegranate. Bioinformatics analysis showed that there were numerous protein insertions and deletion sites in PgARF6b and PgARF6c sequence, and the tertiary structure of PgARF6 proteins were different, which might manifest function differentiation for *PgARF6s*. Protein interaction analysis showed that ARF6 directly interacted with AUX, IAA, and BEE2 to be involved in BR signal. The expression pattern of *PgARF6s* in pomegranate different organs showed that *PgARF6s* were involved in pomegranate flower development. Our results verified that PgARF6a, PgARF6b and PgARF6c proteins were localized in cell nucleus, which were consistent with the localization of CsARF and SlARF (Wu et al., 2011; Xu et al., 2016), indicating that PgARF6s were performed its functions in cell nucleus.

### Expression Patterns of *PgARF6s* in Different Stages and Response to Hormone Treatments

Gene expression analysis from previous studies indicated that ARFs regulate numerous auxin-related processes at different plant developmental stages (Hu et al., 2015; Yu et al., 2020). Su et al.’s (2017) results suggested that *AcoARF3* and *AcoARF11*, the homologous gene of *AtARF6*, might be involved ovule development in pineapple fruit. The expression level of *ARF6* was higher than that of other *FvARFs* in each developing stage of strawberry fruit (Wang et al., 2019). *PgrARF1* and *PgrARF2* were highly expressed in both inner and outer pomegranate seed coat, inferring that these might involve in seed coat development through cell divisions in response to auxin regulation (Yu et al., 2020). The mRNA expression results demonstrated that the expression levels of *PgARF6s* in bisexual flowers were higher than in functional male flowers at the P2 stage of flower development. At the critical stages of ovule sterility P3–P5 and P7–P8 stages of flower organ maturation, *PgARF6s* expression levels in bisexual flowers were lower than that in functional male flowers, indicating that *PgARF6s* were related to ovule sterility and male flower organ maturation. Our results were consistent with the regulation of female sterility in tomatoes by ARF6-miR167 (Nagpal et al., 2005). In addition, we inferred that *PgARF6* might be a negative regulator of ovule development in pomegranate.

*ARF6* and *ARF8* transcriptions are indeed suppressed by exogenously applied IBA (Zhang et al., 2017). In sweet orange, the expression of *CiARF6* genes was markedly down-regulated just after IAA treatment (Li et al., 2015). *PgARF6s* showed different expression patterns under the treatment of exogenous hormones. During the development of bisexual flowers, the expression levels of *PgARF6a* and *PgARF6c* in the 6-BA treatment were lower than that in the control at the critical stages of ovule development (5.1–14.0 mm, P2–P5). During the development of functional male flowers, the transcriptional levels of *PgARF6a* and *PgARF6c* in the 6-BA treatment were lower than that in the control at the critical stages of ovule development (5.1–12.0 mm, P2–P4). Under 6-BA treatment, *PgARF6b* transcriptional level in bisexual flowers was higher than that in functional male flowers at the P2 stage, whereas *PgARF6b* expression level in bisexual flowers were higher than that in functional male flowers at the P3–P5 stage. Under IBA treatment, the transcriptional level of *PgARF6b* in the treatment was lower than that in the control at the critical stages of ovule development (3.0–12.0 mm, P1–P4). During the development of bisexual flowers, the expression levels of *PgARF6a* and *PgARF6c* in the IBA treatment were lower compared with that in the control. During the development of functional male flowers, the expression levels of *PgARF6a* in the IBA treatment were lower than that in the control at the stages of ovule development (3.0–12.0 mm, P1–P4), and the expression levels of *PgARF6c* in the IBA treatment were lower compared with that in the control. Under the 6-BA treatment, *DnARF6* was weakly down-regulated in *D. officinale* seedlings, whereas *DnARF6* was significantly reduced by the IAA treatment (Chen Z. H. et al., 2017). These results indicated that the expressions of *PgARF6a*, *PgARF6b*, and *PgARF6c* were inhibited by the exogenous hormone treatment at the critical stages of ovule development (5.1–12.0 mm, P2–P4), indicating that the expressions of *PgARF6* genes were response to exogenous hormone treatments during the development of pomegranate flowers.

### PgmiR167 Targets PgARF6s Expression in Pomegranate Flowers Development

In *Arabidopsis thaliana*, miR167 and target genes *ARF6* and *ARF8* regulate pistil and stamen development and maturity (Wu et al., 2006). *ARF6* and *ARF8* are essential regulators for ovule and anther development. The flower organs of *arf6 arf8* double mutants show specific phenotypes, such as small petals, closed buds, and short stamens (Nagpal et al., 2005). MiR167 is involved in regulating ovule and stamen development by regulating the expression of *ARF6* and *ARF8* (Ru et al., 2006; Wu et al., 2006). In previous research, down-regulation of *ARF6* and *ARF8* by miR167 leads to floral development defects and female sterility in tomato (Liu N. et al., 2014). In this study, we found that PgmiR167 had only one target binding site on the mature mRNA sequence of *PgARF6s*. The *ARF8*, but not *ARF6*, mRNAs are degraded in the *35S-miR167b* transgenic plants (Ru et al., 2006). The analysis of transcriptome and small-RNA sequencings results showed that *PgARF6a* and PgmiR167a had a directly negative targeted regulation relationship. In addition, we also found that *PgARF6c* expression level was significantly higher than *PgARF6a* and *PgARF6b* in the floral organ development of pomegranate. However, there was no clear the targeted regulation relationship between *PgARF6c* and PgmiR167, suggesting that there may be specific functions for *PgARF6c*. Increased miR167 expression in the transgenic tomato plants lead to reduced transcriptional abundance of *ARF6* and *ARF8* (Liu N. et al., 2014). The results were similar to the previous reports (Wu et al., 2006; Yao et al., 2019), suggesting that the targeting relationship between miR167 and ARF6 was conserved in plants.

### Response of Endogenous Hormone in Pomegranate Flowers to Exogenous Hormone Treatments

Plant hormones are essential signaling molecules that regulate various aspects of plant growth and development. Recent studies have indicated cytokinin in processes related to plant reproductive development and embryogenesis (Šimášková et al., 2015; Cucinotta et al., 2016; Hallmark and Rashotte, 2019). Auxin, BR, GA, and cytokinin are crucial for seed development, and numerous genes regulating seed size are correlated with BRs and auxin (Hu et al., 2018; Zhang X. F. et al., 2020). ABA and ethylene play roles in fruit development and maturation (Fenn and Giovannoni, 2021). Several recent studies suggest that interaction or balance of plant hormones regulate the development of flower buds (Sun et al., 2017; Zhao et al., 2021). Zu et al. (2021). The interaction of BR and cytokinin promotes ovule initiation and increases seed number per silique in *Arabidopsis* (Zu et al., 2021). Hormones are involved in plant development by affecting the expression levels of ARF genes in different plant species (Ha et al., 2013; Chen Z. H. et al., 2017). Our results showed that exogenous plant growth regulators affected the levels of endogenous hormones and *PgARF6* genes expression during pomegranate flower differentiation. *ARFs* in sweet orange and peach positively responded to exogenous auxin but display distinct expression patterns (Li et al., 2015; Diao et al., 2020). The expressions of *PgARF6* genes were inhibited under exogenous 6-BA and IBA treatments, which were consistent with the result of previous research (Chen Z. H. et al., 2017), indicating that *PgARF6s* expression were responsive to exogenous hormones IBA and 6-BA. At the critical stage of ovule sterility (5.1–14.0 mm, P2–P5), exogenous IBA promoted endogenous IAA and ZR accumulation, but inhibited BR. Both exogenous IBA and 6-BA promoted endogenous GA accumulation, and exogenous 6-BA promoted endogenous IAA accumulation at the critical stage of pomegranate ovule development. In the flower bud differentiation stage of “Mantianhong” × “Dangshansu pear” hybrid offspring, the contents of ZT and ABA were increased, but the contents of GA_3_ and IAA were decreased by spraying 100 mg/kg 6-BA (Liu et al., 2015). After 6-BA treatment, the content of IAA in the physiological differentiation stage of apple flower buds was significantly lower than that in the control (Li et al., 2015). After IAA treatment, the contents of endogenous IAA and GAs significantly increased during the physiological differentiation of “Fuji” apple flower buds (Li, 2015). This research was consistent with our result, indicating that exogenous auxin promoted endogenous IAA and cytokinin accumulation in floral organ of plant.

### *PgARF6s* Expression Coordinated Endogenous Hormones Response to Exogenous Hormone Treatments in Pomegranate Flower Development

The results of correlation analysis showed that the content of endogenous hormones was negatively correlated with the expression levels of *PgARF6s* during the development of pomegranate flowers. Under exogenous 6-BA treatment, JA and *PgARF6* genes were grouped together, and JA was negatively correlated with ZR, BR, ABA, and GA. JA content was positively correlated with the expression levels of *PgARF6a* and *PgARF6c*, indicating a significant correlation between the content of JA and *PgARF6s* expressions in the pomegranate flower development. The above results indicated that the interaction between endogenous hormones and genes expressions was jointly involved in regulating the development of pomegranate flower.

**FIGURE 13:**
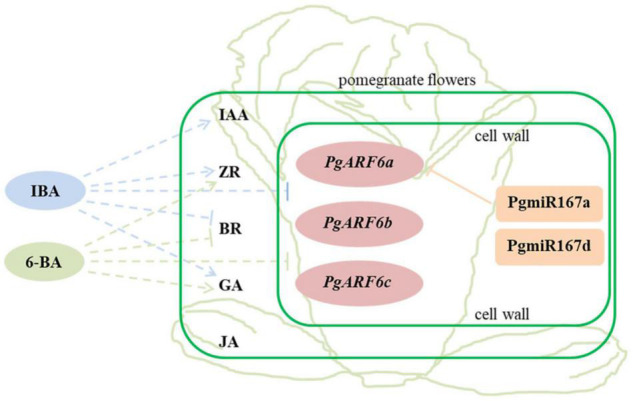
The relationship model for hormones, PgARF6 genes and PgmiR167 in pomegranate flowers development. Solid line represents the direct relationship, dotted line represents the indirect relationship; arrow represents promotion and vertical line represents inhibition.

In our study, three *PgARF6* genes have the same gene structure and conserved domains. *PgARF6s* expression levels in bisexual flowers were significantly lower than those in functional male flowers at the critical stage of ovule sterility (8.1–14.0 mm), indicating that *PgARF6* genes had the conservative function of involving in ovule sterility developing into functional male flower. However, numerous differences were found in PgARF6 proteins sequences and tertiary structure. *PgARF6s* expression patterns were different in pomegranate flower and under exogenous hormones treatment, the expression of *PgARF6c* was mightily inhibited by the exogenous hormone, and *PgARF6a* was directly regulated by PgmiR167a, indicating that *PgARF6* genes performed different function in pomegranate flower development. Based on experimental data, we proposed a relationship model for hormones, *PgARF6* genes and PgmiR167 in pomegranate flowers (Figure 13). PgARF6s functioned in cell nucleus, and PgmiR167a directly targeted *PgARF6a* and affected its expression. Under hormone treatment, the expressions of three *PgARF6* genes were inhibited by exogenous IBA and 6-BA. At the critical stage of ovule sterility (8.1–12.0 mm), exogenous IBA promoted the accumulation of IAA, GA, and ZR, and reduced the accumulation of BR. When the vertical diameter was 8.1–12.0 mm, exogenous 6-BA promoted the content of ZR and GA accumulation, and inhibited BR accumulation. Under 6-BA treatment, ZR accumulation was negatively correlated with the expressions of *PgARF6* genes. Under IBA treatment, the content of BR, IAA, and JA were negatively correlated with the expressions of *PgARF6* genes. Our results suggested that *PgARF6* genes were involved in ovule sterility of pomegranate flowers.

## Data Availability Statement

The datasets presented in this study can be found in online repositories. The names of the repository/repositories and accession number(s) can be found in the article/Supplementary Material.

## Author Contributions

YZ and ZY conceived and planned all experiments. YZ analyzed and cloned *PgARF6* subfamily gene members and wrote the manuscript. YZ and YW performed the expression level examination. YZ and YR helped to prepare datasets and figures for the manuscript. YZ and MY were the major contributors in analyzing data. XZ and ZY revised the manuscript. All authors read and approved the final manuscript.

## Conflict of Interest

The authors declare that the research was conducted in the absence of any commercial or financial relationships that could be construed as a potential conflict of interest.

## Publisher’s Note

All claims expressed in this article are solely those of the authors and do not necessarily represent those of their affiliated organizations, or those of the publisher, the editors and the reviewers. Any product that may be evaluated in this article, or claim that may be made by its manufacturer, is not guaranteed or endorsed by the publisher.

## Footnotes

1
https://www.ncbi.nlm.nih.gov/cdd

2
http://smart.embl-heidelberg.de//

3
http://gsds.cbi.pku.edu.cn

4
https://meme-suite.org/meme/tools/meme

5
https://www.omicstudio.cn/tool/53

6
http://www.csbio.sjtu.edu.cn/bioinf/Cell-PLoc-2/
