# Supplementary material for: ARF6s Identification and Function Analysis Provide Insights Into Flower Development of Punica granatum L
Source: Front Plant Sci. 2022 Mar 7;13:833747. doi: 10.3389/fpls.2022.833747 (PMC8937018; doi:10.3389/fpls.2022.833747)
Supplement: Supplementary file 1 [file Data_Sheet_1.docx]

**Supplementary Information**

**Title: ARF6s identification and function analysis provide insights into flower development of *Punica granatum* L.**

Supplemental Figure S1 Gene cloning of *PgARF6s*


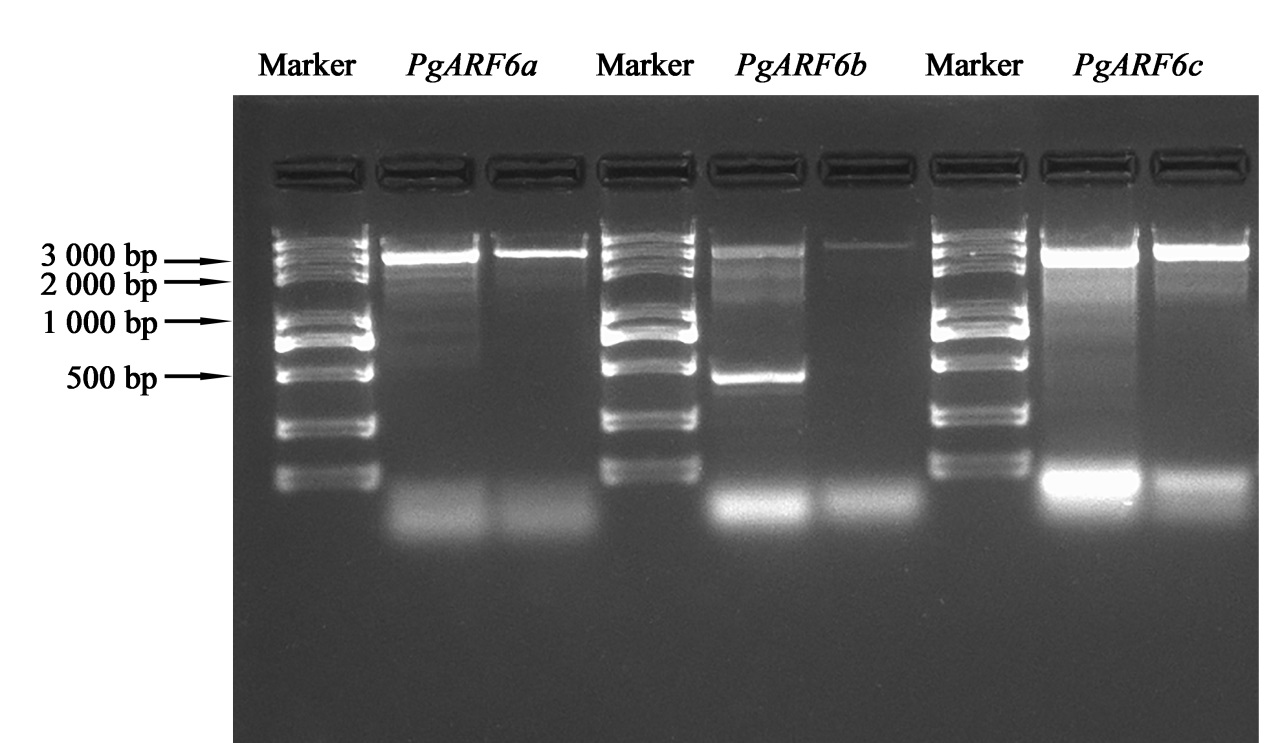


Supplemental Figure S2 Protein-protein interaction network of ARF6


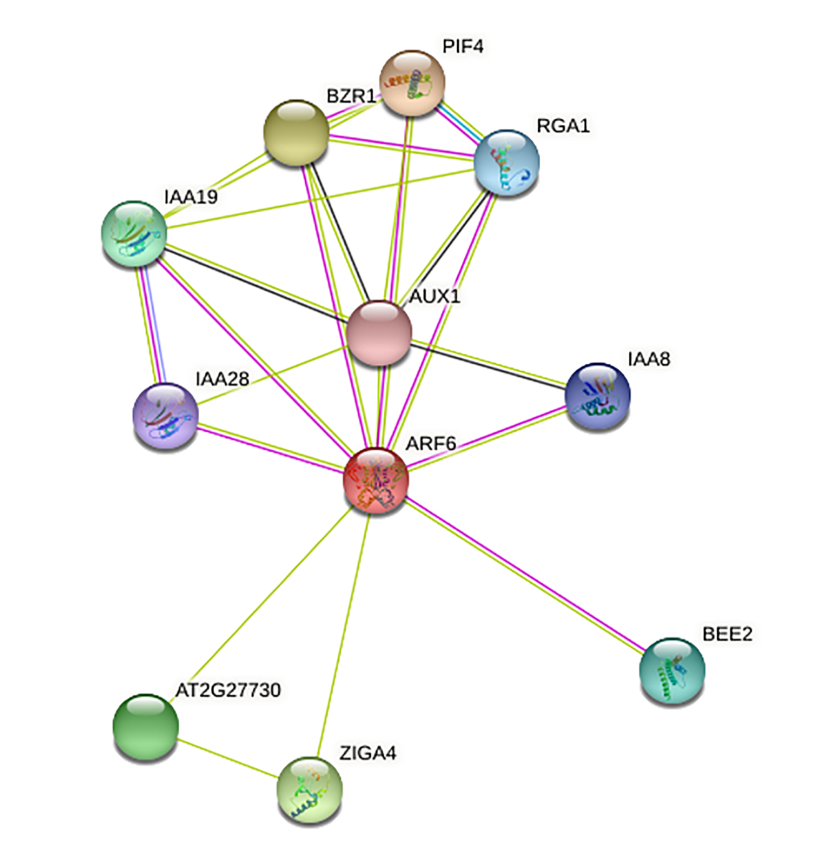


Note: Red participates in hormone-mediated signalling pathway; Blue participates in auxin-activated signalling pathway; Green participates in biological regulation or negative regulation of development process; Yellow participates in plant organ development or floral whorl development; Purple participates in flower development or floral organ development.

Supplemental Figure S3 Multiple sequences alignment of ARF6 in different species


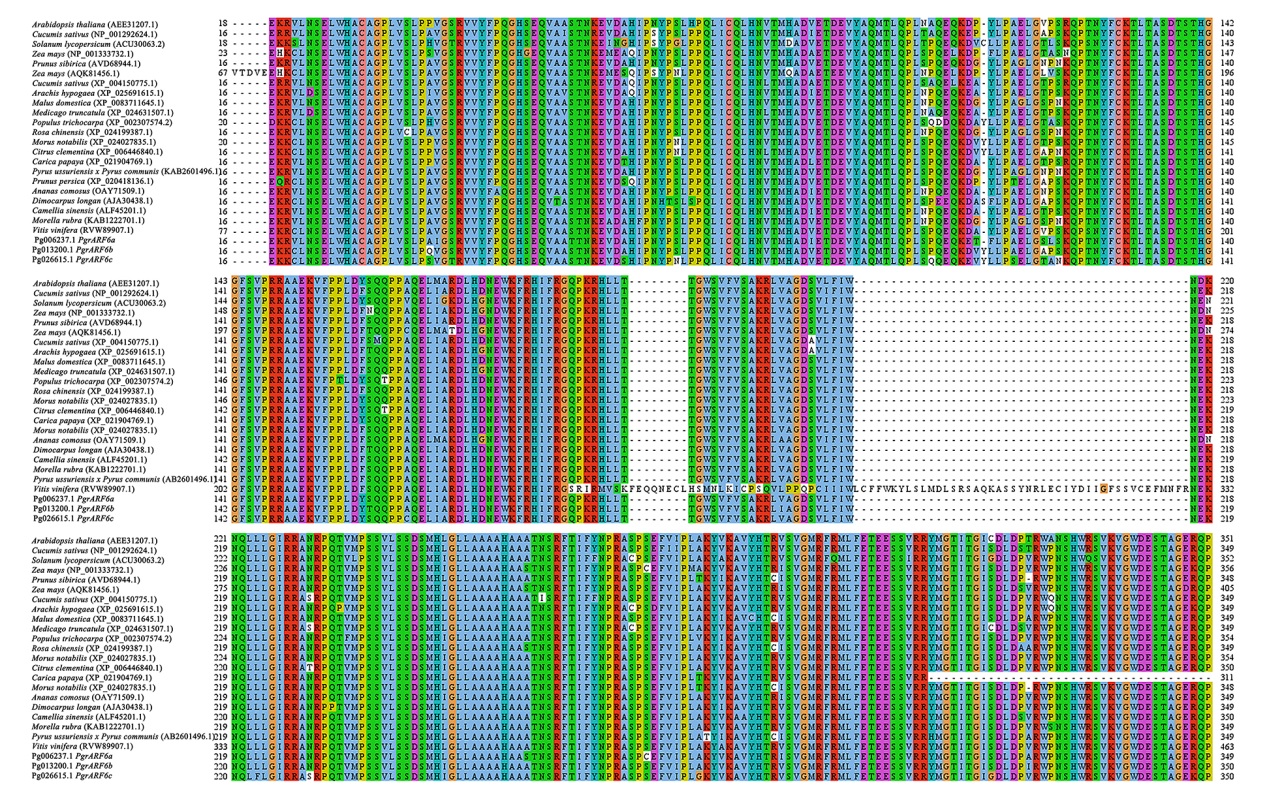


Supplemental Figure S4 Phylogenetic relationship among ARF6 proteins from different species


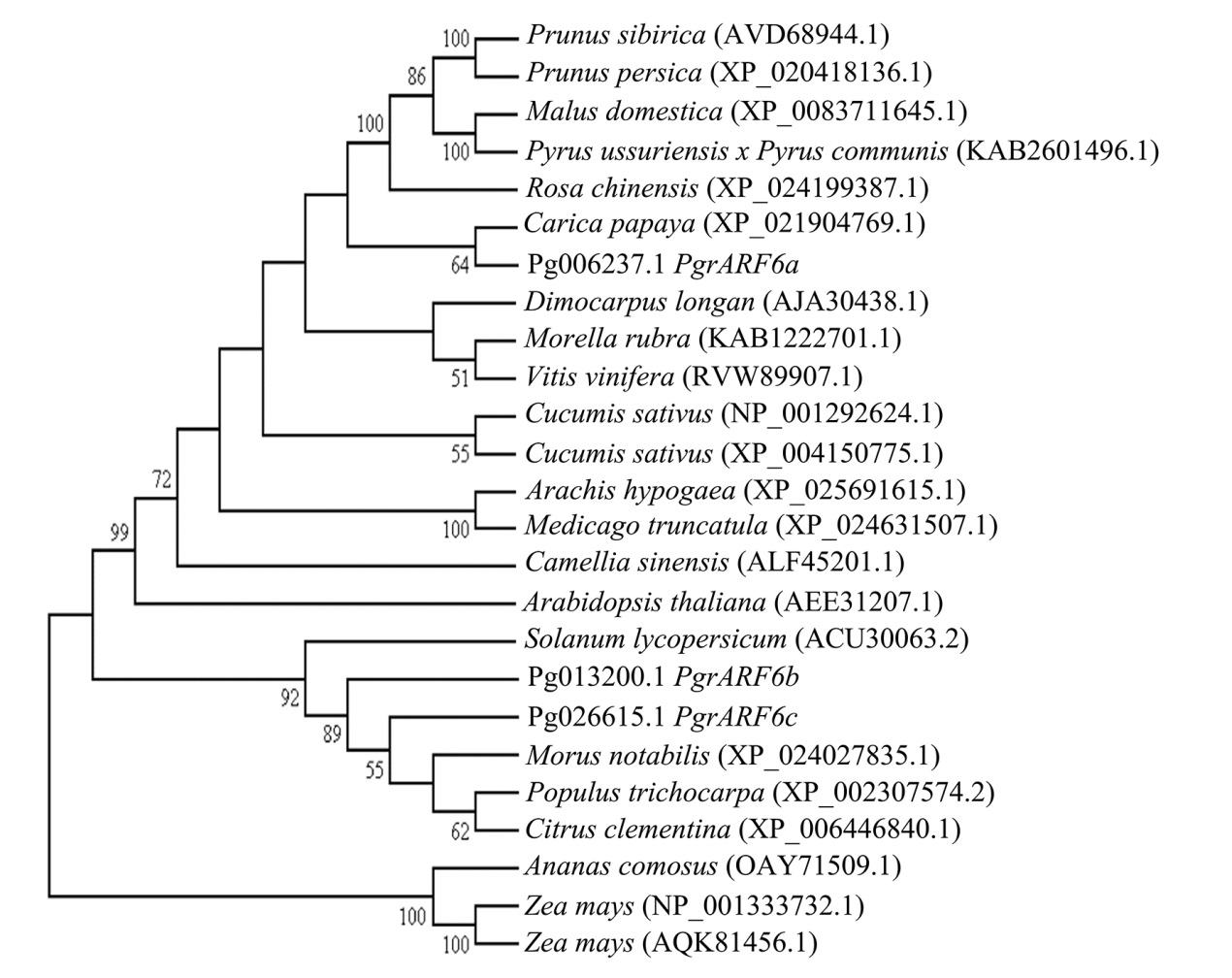


Supplementary Table S1 RNA-Seq data of pomegranate

| Accession No. | Cultivars | Sample type | Platform | Note |
| --- | --- | --- | --- | --- |
| SRR5279396 | ‘Dabenzi’ | Root | Illumina HiSeq 4000 |  |
| SRR5279397 | ‘Dabenzi’ | Leaf | Illumina HiSeq 4000 |  |
| SRR5279395 | ‘Dabenzi’ | Flower | Illumina HiSeq 4000 |  |
| SRR5279391 | ‘Dabenzi’ | Inner seed coat (50 days after pollination) | Illumina HiSeq 4000 |  |
| SRR5279388 | ‘Dabenzi’ | Outer seed coat (50 days after pollination) | Illumina HiSeq 4000 |  |
| SRR5279394 | ‘Dabenzi’ | Pericarp (50 days after pollination) | Illumina HiSeq 4000 |  |
| SRR5446596-SRR5446598 | ‘Tunisia’ | 3.0-5.0 mm flower | Illumina HiSeq 2500 | Bisexual flower |
| SRR5446593-SRR5446595 | ‘Tunisia’ | 5.1-13.0 mm flower | Illumina HiSeq 2500 | Bisexual flower |
| SRR5446590-SRR5446592 | ‘Tunisia’ | 13.1-25.0 mm flower | Illumina HiSeq 2500 | Bisexual flower |
| SRR5446605-SRR5446607 | ‘Tunisia’ | 3.0-5.0 mm flower | Illumina HiSeq 2500 | Functional male flower |
| SRR5446602-SRR5446604 | ‘Tunisia’ | 5.1-13.0 mm flower | Illumina HiSeq 2500 | Functional male flower |
| SRR5446599-SRR5446601 | ‘Tunisia’ | 13.1-25.0 mm flower | Illumina HiSeq 2500 | Functional male flower |
| SRR5678820 | ‘Tunisia’ | Inner seed coat (50 days after pollination) | Illumina HiSeq 4000 |  |
| SRR5678819 | ‘Baiyushizi’ | Inner seed coat (50 days after pollination) | Illumina HiSeq 4000 |  |

Supplementary Table S2 Information of ARF gene family in ‘Taishanhong’ pomegranate

| Gene name | Gene ID | Location | CDS (bp) | Exon No. | Amino acid residues | Strand | MW(Da) | PI |
| --- | --- | --- | --- | --- | --- | --- | --- | --- |
|  | Pg016028.1 | scaffold3:3932763:3936927 | 2415 | 13 | 804 | - | 89781.57 | 6.13 |
|  | Pg018864.1 | scaffold38:1163589:1169845 | 3345 | 13 | 1114 | - | 124541.30 | 6.50 |
|  | Pg024734.1 | scaffold6:436649:441697 | 3048 | 12 | 1015 | + | 111594.67 | 6.77 |
| *PgARF6b* | Pg013200.1 | scaffold22:3155748:3161904 | 2901 | 14 | 966 | + | 107094.91 | 6.35 |
|  | Pg026615.1 | scaffold7:348437:354121 | 2658 | 14 | 885 | - | 98364.71 | 6.12 |
|  | Pg026370.1 | scaffold7:3711215:3720981 | 2301 | 17 | 766 | - | 84195.65 | 6.33 |
| *PgARF6a* | Pg006237.1 | scaffold14:2389996:2396883 | 2649 | 14 | 882 | - | 97086.48 | 6.03 |
| *PgARF6c* | Pg022651.1 | scaffold5:1520371:1525194 | 2844 | 15 | 947 | + | 104431.37 | 5.54 |
|  | Pg013270.1 | scaffold22:4248531:4251107 | 2073 | 3 | 690 | + | 75777.88 | 8.32 |
|  | Pg019680.1 | scaffold4:452421:456110 | 2049 | 14 | 682 | + | 76427.42 | 6.24 |
|  | Pg013965.1 | scaffold239:81974:86289 | 1698 | 10 | 565 | - | 63016.04 | 8.44 |
|  | Pg021268.1 | scaffold44:677607:682384 | 2355 | 12 | 784 | + | 85831.65 | 6.04 |
|  | Pg029792.1 | scaffold9:1482448:1486007 | 2055 | 14 | 684 | - | 76670.57 | 5.85 |
|  | Pg028775.1 | scaffold81:175373:178083 | 2049 | 4 | 682 | + | 74707.83 | 8.86 |
|  | Pg030661.1 | scaffold97:543357:546732 | 1962 | 8 | 653 | - | 72880.76 | 7.65 |
|  | Pg026209.1 | scaffold69:647074:651969 | 2070 | 10 | 689 | - | 75466.76 | 6.74 |
|  | Pg015343.1 | scaffold27:1815147:1817644 | 2031 | 3 | 676 | + | 74457.03 | 6.77 |
|  | Pg028784.1 | scaffold81:294340:298542 | 2307 | 12 | 768 | + | 85121.10 | 6.15 |
|  | Pg002408.1 | scaffold109:636915:639514 | 1707 | 2 | 568 | - | 61601.33 | 5.71 |
